# Protective roles of peroxiporins AQP0 and AQP11 in human astrocyte and neuronal cell lines in response to oxidative and inflammatory stressors

**DOI:** 10.1042/BSR20231725

**Published:** 2024-03-22

**Authors:** Zein Amro, Lyndsey E. Collins-Praino, Andrea J. Yool

**Affiliations:** School of Biomedicine, University of Adelaide, Adelaide, SA 5005, Australia

**Keywords:** aquaporins, hydrogen peroxide, lipopolysaccharides, neurodegeneration, protein colocalization

## Abstract

In addition to aquaporin (AQP) classes AQP1, AQP4 and AQP9 known to be expressed in mammalian brain, our recent transcriptomic analyses identified *AQP0* and *AQP11* in human cortex and hippocampus at levels correlated with age and Alzheimer’s disease (AD) status; however, protein localization remained unknown. Roles of AQP0 and AQP11 in transporting hydrogen peroxide (H_2_O_2_) in lens and kidney prompted our hypothesis that up-regulation in brain might similarly be protective. Established cell lines for astroglia (1321N1) and neurons (SHSY5Y, differentiated with retinoic acid) were used to monitor changes in transcript levels for human AQPs (*AQP0 to AQP12*) in response to inflammation (simulated with 10–100 ng/ml lipopolysaccharide [LPS], 24 h), and hypoxia (5 min N_2_, followed by 0 to 24 h normoxia). *AQP* transcripts up-regulated in both 1321N1 and SHSY5Y included *AQP0, AQP1* and *AQP11*. Immunocytochemistry in 1321N1 cells confirmed protein expression for AQP0 and AQP11 in plasma membrane and endoplasmic reticulum; AQP11 increased 10-fold after LPS and AQP0 increased 0.3-fold. In SHSY5Y cells, AQP0 expression increased 0.2-fold after 24 h LPS; AQP11 showed no appreciable change. Proposed peroxiporin roles were tested using melondialdehyde (MDA) assays to quantify lipid peroxidation levels after brief H_2_O_2_. Boosting peroxiporin expression by LPS pretreatment lowered subsequent H_2_O_2_-induced MDA responses (∼50%) compared with controls; conversely small interfering RNA knockdown of AQP0 in 1321N1 increased lipid peroxidation (∼17%) after H_2_O_2_, with a similar trend for AQP11 siRNA. Interventions that increase native brain peroxiporin activity are promising as new approaches to mitigate damage caused by aging and neurodegeneration.

## Introduction

The thirteen classes of mammalian aquaporin channels (AQP0 to AQP12), known for diverse roles in fluid transport, show tissue specific patterns of expression throughout the body [1]. In the human brain, AQP1, AQP4 and AQP9 have been well characterized [2–6]. AQP1 channels in the choroid plexus facilitate cerebrospinal fluid production [2] and are up-regulated in reactive astrocytes in pathological conditions including Alzheimer’s disease (AD) [3]. Abundantly expressed AQP4, known as the ‘brain AQP’ [4], is located in the perivascular end-feet of astrocytes, involved in regulating cell volume, mediating water exchange across blood–brain barrier and through the glymphatic system [4,5,7,8]. The role of AQP4 in the clearance of pathological tau from the interstitial fluid was supported by studies of *aqp4*-null mice, in which impaired clearance was associated with pathology reminiscent of AD [7,9,10]. AQP9 in astrocytes is localized in cell bodies and thought to be involved in energy metabolism [11,12], including handling of lactate and glycerol, which are also affected in aging and AD [6,13].

The aim of this project was to investigate roles for two unexpected classes of AQPs (*AQP0* and *AQP11*) recently found by our group to be expressed at the transcript level in human brain [14] but not yet assessed for protein localization or possible functional roles in human neuronal and glial cells. The levels of transcript of the novel brain AQPs correlated with patient age and AD status [14]; however, one limitation of the RNAseq approach was that signal localization in neurons and glia, and possible dynamic changes in AQP expression remained unknown. To address this gap, work here evaluated the patterns of transcript expression of the human *AQPs* in neuronal and astroglial cell lines under baseline and stressed conditions, assessed protein levels and subcellular localization of the two novel subtypes AQP0 and AQP11, and evaluated the capacity of these channels to protect cells against stressor-induced damage. Precedent for a protective role of AQP11 was documented by Atochina-Vasserman and colleagues with a comprehensive analysis done in mouse kidney [15]. Their results showed AQP11 is highly expressed in the endoplasmic reticulum of kidney proximal tubular cells where it is required for preventing oxidative stress-induced damage during periods of high glucose-induced metabolic activity, as revealed by the proximal tubule apoptosis, mitochondrial loss, kidney damage and kidney failure seen in mice genetically deficient in wild-type AQP11 [15]. Both protein and transcript levels for AQP11 in kidney cells were up-regulated in response to exposure to increased glucose, promoting protection from the effects of metabolically generated reactive oxygen species; the protective effect was compromised after siRNA-knockdown of AQP11 levels, and suggested interesting potential clinical relevance to diabetic patients with chronic kidney disease who might carry *AQP11* sequence polymorphisms [15]. Work here identifies a comparable protective role for AQP11 channels in neural and glial cell types, and suggests that the clinical importance of peroxiporins extends across multiple organ systems, thus presenting an attractive novel candidate for therapeutic approaches aimed at up-regulation of peroxiporin expression (or channel activity) to boost innate protective cell defences in tissues with high metabolic demands.

The structure of the aquaporin protein is a membrane spanning channel, with four subunits organized into a tetramer, confirmed by crystal structure analyses [16]. Intrasubunit pores allow permeation of water as well as other substrates such as glycerol and H_2_O_2_, depending on the subtype, and a central pore in the middle of the tetramer which has been proposed in some classes of AQPs to serve as a pathway for fluxes of gases or charged particles such as ions; these concepts have been covered in prior reviews [17–20]. Subtypes of AQPs have been classified by functional properties and amino acid sequence into different categories [18]. The classical AQPs (AQP0, AQP1, AQP2, AQP4 and AQP5) allow transmembrane fluxes of water through selective pores; additional permeabilities to metabolites, ions and other substrates have been demonstrated as well [21,22]. The aquaglyceroporins (AQP3, AQP7, AQP9 and AQP10) are permeable to glycerol and water [23], as well as lactate in AQP9 [24]. The peroxiporins are a subset of AQPs which enable H_2_O_2_ permeability, including AQP0 and AQP5 in the eye, and AQP1, AQP6, AQP8 and AQP11 in other organs [25–28]. Recent transcriptomic analyses of the Allen Brain Atlas database by our group revealed that additional classes of AQPs, including peroxiporins (*AQP0, AQP5, AQP8, AQP11*) and aquaglyceroporins (*AQP3, AQP7, AQP10*), are expressed in the human brain [14], extending prior work which showed a subset of these were present in the rat brain [29,30]. Aquaporin functions and mechanisms of regulation show an impressive diversity consistent with the appearance of this ancient class of channels early during evolutionary time, and subsequent diversification and radiation into hundreds if not thousands of classes that enable a broad array of physiological processes essential across all the major divisions of life [31–34].

Neuroinflammation and hypoxia are features of stroke [35,36], brain cancers [37,38] and neurodegenerative disorders [39,40] that increase oxidative stress and worsen pathological outcomes [41,42]. Neuroinflammation activated by the bacterial endotoxin lipopolysaccharide (LPS)^40^ and hypoxia, defined as a 2–5% fall in oxygen (below arterial saturation level) [43], were shown in previous studies to increase AQP4 expression in astrocytes [44,45]; but possible effects on the expression of other AQPs remained unexplored. We hypothesized that neuronal and glial responses to stressors could involve the novel peroxiporin classes. Stress-induced regulation of AQP transcript and protein levels was examined using differentiated cholinergic neurons produced from human SHSY5Y neuroblastoma cells (maturation induced by 7-day supplementation with retinoic acid) [46]; and human 1321N1 astrocytoma cells as a model for brain astroglia [47]. Outcomes here are the first to demonstrate that neuronal and glial cell lines show differential stressor-induced patterns of regulation and distinctive patterns of peroxiporin localization under stress treatment conditions.

For years, hallmarks of AD (tau protein, neurofibrillary tangles, amyloid plaques) which are known to correlate with declining cognitive abilities have captured the research spotlight, but linked treatments have shown poor translation to the bedside [48–52], with critical questions raised about efficacy and side-effect profiles. New treatment options aimed at other components of the pathophysiological cascade are critically needed. Oxidative stress caused by accumulated H_2_O_2_ is emerging as one underestimated neuropathogenic factor [53], prompting our proposal that boosting native cell mechanisms for handling H_2_O_2_ could be invaluable as a novel AD therapy. Our findings have relevance to medical practice in advancing a new concept, launching a search for treatments that act on peroxiporins channel expression or functional activity in order to slow the deleterious effects of aging and neurodegenerative disease by augmenting native protective mechanisms.

## Materials and methods

### Cell culture

The human SHSY5Y neuroblastoma cell line (Sigma, Australia), originally a sub-line from SK-N-SH bone marrow cells, was utilized to culture neuroblastoma cells that acquired cholinergic neuronal properties after differentiation *in vitro* [54]. The culture medium consisted of 1:1 Dulbecco’s Modified Eagle Medium (DMEM) and Ham’s F12 supplemented with 10% (V/V) Fetal Bovine Serum (FBS), 1% GlutaMAX (200 mM; V/V) and 1% penicillin and streptomycin (5000 U/ml; V/V). To drive differentiation, cells at 80–90% confluency were starved by reducing the percentage of FBS from 10% to 1% in the medium to inhibit pluripotency, and by repeated supplementation with 10 µM all-trans retinoic acid (ATRA; Sigma, Australia) at days 1, 3 and 5 to evoke differentiation into neurons [46], as confirmed by cholinesterase activity and neural marker expression. The human 1321N1 astrocytoma cell line (Sigma, Australia) originally sub-cloned from the 1181N1 cell line isolated from malignant glioma U-118 cells, provided cultured cells with previously demonstrated astrocyte properties [55]. BV2 murine cells (kindly provided by Prof M Hutchinson, Adelaide, Australia) were cultured as a model for microglia [56] and were included for comparison cell line in the malondialdehyde cell viability assays. Astroglial cells were cultured in full DMEM supplemented with 10% FBS, 1% GlutaMAX and 1% penicillin and streptomycin. All lines were incubated at 37°C with 5% CO_2_. All were seeded at ∼200,000 cells per 2 ml in 6-well plates for all experiments except immunocytochemistry (ICC). For ICC, ∼3000 cells in 300 µl were seeded on ibidi µ-slide 8 well plates (ibidi 80806, Germany) and incubated for 24 h prior to fixation and staining. Cultures were established from passages 4-10 for 1321N1 cells, and from passages 14-16 for SHSY5Y (the earliest available).

### Cellular stress models

To simulate inflammation *in vitro*, cells were treated with either 10 or 100 ng/ml of LPS (Invitrogen, Australia) for 24 h as per published protocols [57,58] prior to RNA extraction or immunocytochemistry (ICC). To induce hypoxia, cells were placed in a 10 L environmental chamber that was flushed with N_2_ for 5 min, after which cultures were returned to normal oxygen, media was replaced as per established protocols, and cultures were incubated at 37°C [59,60]. Cell plates were then maintained under normal oxygen conditions until RNA extractions were done at 0, 12 and 24 h after return to normoxia.

### Cholinergic enzyme activity

Cholinergic neuron differentiation of the SHSY5Y cells was verified by acetylcholinesterase (AChE) activity as described by Ellman [61]. In brief, total protein was extracted in lysis buffer (20 mM HEPES, 150 mM NaCl, and 1% NP40 with phosphatase inhibitors). Cell lysates were incubated 5 min with 10 mM 2-nitrobenzoic acid (Ellman’s reagent; Sigma), followed with 8 mM acetylthiocholine chloride (Sigma). Yellow signal intensity was used to quantify the breakdown by AChE of acetylthiocholine into thiocholine which reacts with Ellman’s reagent, measured at 412 nm (10 min). Non-differentiated SH-SY5Y neuroblastoma cells served as the negative control for comparison. Data were compiled from four sets of cultured cells with two replicates each per treatment group.

### Quantitative PCR (qPCR)

The mRNA transcript levels of the 13 classes of human aquaporin channels (*AQPs 0-12*) in the two cell lines (1321N1 and differentiated SHSY5Y neurons) were quantified after control and stressor treatment conditions. Total RNA was extracted using the RNeasy mini kit (Qiagen, U.S.A.). After extraction, 1.5 µg total RNA was used for cDNA synthesis (QuantiTect Reverse Transcription Kit). Approximately 100 ng/µl of cDNA (assessed by Nanodrop Spectophotometer; ThermoFisher, Australia) was used for each qPCR reaction, carried out by 2-step analysis with the KAPA SYBR™ FAST qPCR Master Mix (Sigma, Australia) in a 10 µl volume with 10 µM primers for *AQP0* to *AQP12* (Supplementary Table S1) and for reference genes β-actin (neurons) and GAPDH (astrocytes). All data were standardized to the reference genes using the 2-ΔCT protocol [62]. Data shown are the relative expression levels referenced to corresponding control levels, compiled as histograms. Data were compiled from four sets of cultured cells with two replicates each per treatment group.

### Immunocytochemistry (ICC)

The localization of AQP0 and 11 channels in astrocytes and neurons with and without LPS stimulation was determined with immunocytochemistry (ICC). Samples of 500 cells in 300 µl of culture medium in 8-well IBIDI µ-slides were fixed with 1:1 acetone and methanol for 15 min at room temperature, followed by three rinses in cold phosphate buffer saline (PBS). Cells were blocked in 0.1% TWEEN with 1% bovine serum albumin (BSA) for 1 h, followed by 1 h incubations with rabbit anti-human primary antibodies to AQP0 (Abcam; 1:100, ab15077) or AQP11 (Abcam; 1:100 ab12281). To visualize the possible localization of AQPs in plasma membranes, cells were double-labeled with a mouse anti-human primary antibody to Na^+^-K^+^-ATPase (Abcam; 1:100, ab283318) in combination with rabbit anti-human antibodies to AQP0 or AQP11 for 1 h. Cells were washed three times with cold PBS followed by incubation 1 h with Alexa488-tagged goat anti-mouse secondary antibody (1:1500, Abcam) and Alexa568-tagged goat anti-rabbit (1:1500, ThermoFisher, Australia). Cells were then washed twice with cold PBS and incubated at 37°C with Hoechst nuclear stain (1:1500, Sigma). For endoplasmic reticulum (ER) co-staining, green ER CytoPainter (1:1000) prepared in 1× buffer solution (ER staining kit; ab13948) was applied for 20 min, then rinsed with PBS, before mounting medium was applied in conjunction with Hoechst stain. The Area-Quantification FL V.2.1.3 program on HALO (Indica Labs, New Mexico, U.S.A.) was used to quantify staining intensity, which was averaged and compiled into histograms. Z-stack images were merged to detect colocalization using the analysis software (Imaris, U.K.). All images were captured at 60X magnification using a Confocal Olympus FV3000 microscope (Adelaide Microscopy, University of Adelaide, SA Australia).

### Malondialdehyde (MDA) cell viability assay

To investigate potential roles of AQPs 0 and 11 in oxidative stress responses, total malondialdehyde (MDA) lipid peroxidation levels were quantified using a colorimetric assay (Abcam; ab233471, Australia). Briefly, 1321N1 astrocytes were stressed with 10 or 100 ng/ml LPS for 24 h, lyzed and centrifuged at 13,000 × ***g*** to collect supernatant to which was added 600 µl thiobarbituric acid (TBA) reagent. Subsets of 1321N1 and BV2 cells were additionally subjected to 5 µM of H_2_O_2_ for 5 min immediately before lysis to evaluate effects of oxidative stress; equivalent cells with and without LPS, but not treated with the final H_2_O_2_ pulse served as comparisons. Samples were incubated 1 h at 95°C to allow TBA and MDA to react, producing a yellow product measured at OD_532_. Total MDA levels (nmol/ml) were calculated as per manufacturer’s protocols. Data were compiled from three sets of cultured cells with two replicates each per treatment group.

### AQP small interfering RNA (siRNA) knockdown

Treatment with 100 ng/ml LPS (24 h) was used to stimulate AQP expression in 1321N1 cells. To assess whether up-regulation of AQPs 0 and 11 was associated with increased peroxiporin function, small interfering (si)RNA knockdown experiments were conducted. Two siRNA kits each for AQP0 (Ambion; S8784 & S8786 ThermoFisher, Australia), AQP11 (Ambion; S49052 & S49053, ThermoFisher, Australia) and scrambled negative controls (Ambion; 4390843 & 4390846 ThermoFisher, Australia) were optimized using qPCR to determine that 72 h was maximal for gene silencing (Supplementary Figure S1). The 72 h siRNA incubation period was used for subsequent experimental tests. siRNA transfection media was replaced by fresh medium after 48 h siRNA incubation, and cells were tested at the 72 h timepoint for susceptibility to lipid peroxidation using the MDA assay as described above. siRNA sequences are proprietary information not disclosed by the manufacturer. Data were compiled from three sets of cultured cells with two replicates each per treatment group.

### Statistical analyses

For statistical analyses of all data sets, one-way ANOVA followed by multiple comparisons with the post hoc Tukey test was conducted using GraphPad Prism V9.0 (San Diego, CA). The threshold for statistical significance was set as *P*<0.05.

## Results

### Pattern of AQP expression during SHSY5Y differentiation

**Figure 1:**
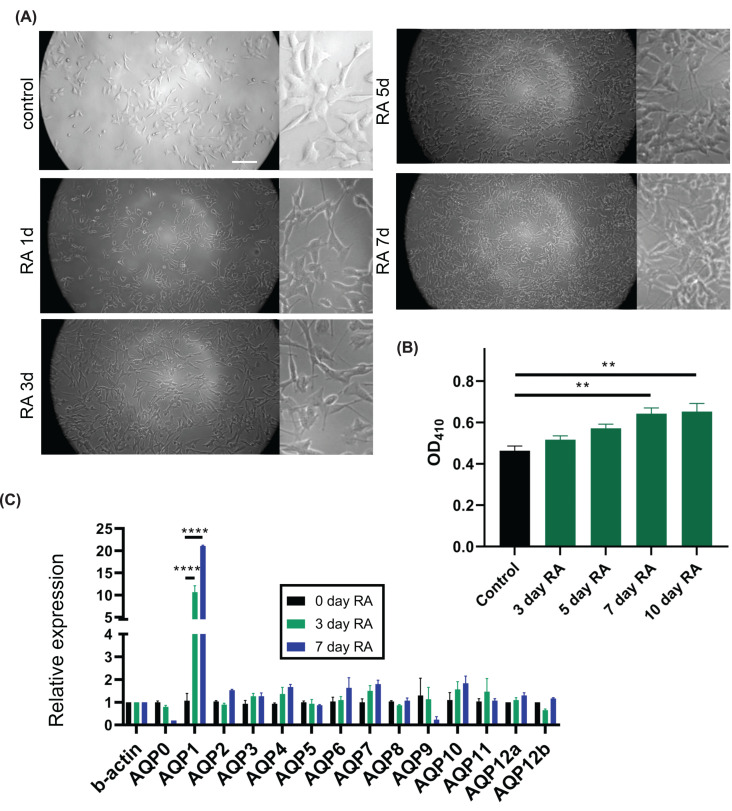
SHSY5Y neuronal differentiation induced by retinoic acid (RA) and effects on *AQP* expression (**A**) Morphological differentiation induced by 10 μM RA at 1, 3, 5 and 7 days of treatment, compared with non-RA treated control. Scale bar is 0.5 mm; insets are 3× (167 µm). (**B**) Colorimetric assessment of increased acetylcholinesterase (AChE) enzymatic activity during retinoic acid (RA) induced differentiation, measured on a plate reader at OD410 (ODU). (**C**) Relative transcript levels of all classes of human *AQP*s (*AQP0* to *AQP12*) in SHSY5Y cells at days 0, 3 and 7 of RA-induced differentiation, normalized to the reference gene, *β-ACTIN*, using the 2-ΔCT calculation method. **P<0.01, ****P<0.0001. Data are mean ± SEM; *n* = 6 per group.

Transcript levels for all *AQP* classes *(AQP0* to *AQP12*) were measured by qPCR in SHSY5Y neuroblastoma cells (Figure 1) during differentiation with retinoic acid (RA). Correct maturation was confirmed by the development of characteristic polarized morphologies and extensive neurite arborizations at days 7–10 *in vitro* (Figure 1A), and by expression of the enzyme acetylcholinestease (a marker for cholinergic neurons), which showed approximately 50% higher activity at days 7 and 10 of RA treatment as compared with non-RA control SHSY5Y cells (Figure 1B). The neuronal phenotype also was supported by the expression of the neuronal marker NeuN by day 7, not seen in non-RA control cells (Supplementary Figure S2). Transcript levels for all *AQP* classes were quantified by qPCR as a function of time during RA-induced differentiation, and showed a marked 10- to 20-fold increase for *AQP1* at days 3–7 of maturation (Figure 1C), without appreciable changes in the transcript levels of any other classes of *AQPs* during the differentiation process.

### LPS stress up-regulates aquaporin transcript levels in SHSY5Y neurons and 1321N1 astrocytes

**Figure 2:**
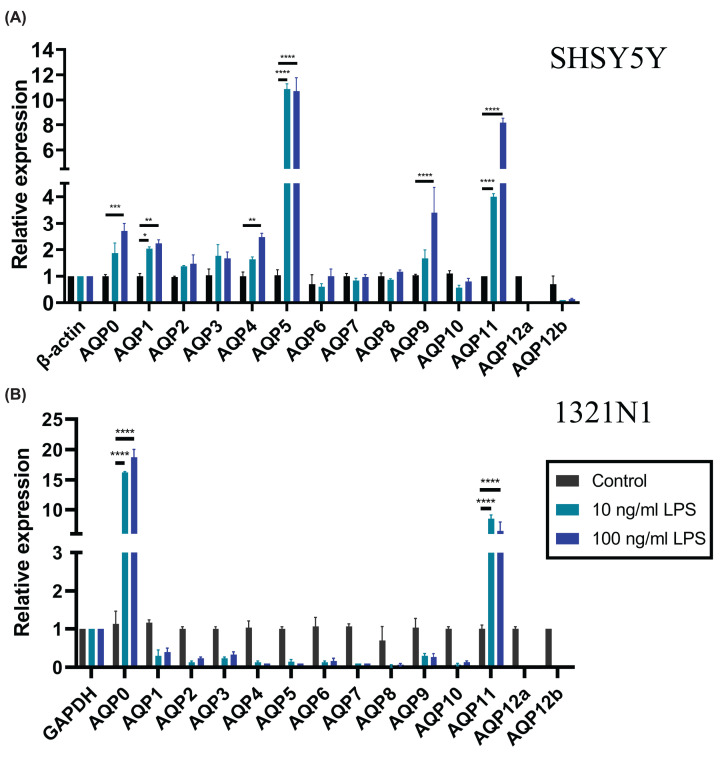
Effects of LPS stress on aquaporin expression patterns in astrocyte and neuronal cell lines Relative RNA levels of human *AQP0* to *AQP12* were assessed after 24 h in control or LPS treatment conditions for: (**A**) SHSY5Y RA-treated neurons at 7 days and (**B**) 1321N1 astrocytes. Levels calculated using the 2-ΔCT method were normalized to the housekeeping genes *GAPDH* for astrocytes, and *ß-ACTIN* for neurons and microglia; *P<0.05, **P<0.01, ***P<0.0001, ****P<0.00001. Data are mean ± SEM; *n* = 6 per group.

Treatment of cultured 1321N1 astrocytes and SHSY5Y neurons at differentiation day 7 with either 10 or 100 ng/ml LPS led to marked increases in aquaporin transcript levels after 24 h (Figure 2). In SHSY5Y neurons, increased transcript levels for *AQP1, AQP5* and *AQP11* were observed after 10 ng/ml LPS treatment. After 100 ng/ml LPS stimulation, increased levels were observed for the same three transcripts (*1, 5* and *11*), as well as *AQP0, AQP4* and *AQP9* (Figure 2A). Cultured 1321N1 astrocytes showed a more focused profile of expression after LPS stress, with only *AQP0* and *AQP11* showing up-regulation at either 10 or 100 ng/ml LPS (Figure 2B). In summary, both *AQP0* and *AQP11* were up-regulated under LPS stress in cell models for neurons and astrocytes. Possible down-regulation of other *AQP*s by LPS in 1321N1 astrocytes cannot be ruled out, but was not statistically significant under the conditions tested.

### Hypoxic stress up-regulates aquaporin transcript levels in SHSY5Y neurons and 1321N1 astrocytes

**Figure 3:**
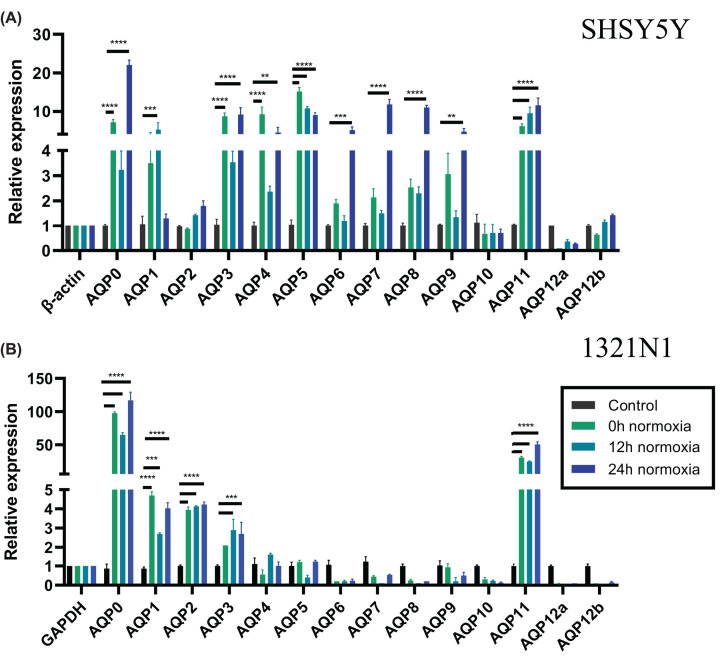
Effects of hypoxic stress on aquaporin expression patterns in astrocyte and neuronal cell lines Relative RNA levels of human *AQP0* to *AQP12* were assessed after 1 h hypoxia followed by 0, 12 and 24 h normoxia in (**A**) SHSY5Y RA-treated neurons at day 7 and (**B**) 1321N1 astrocytes. Control cells received no hypoxic stress. Expression was normalized to the reference gene *GAPDH* for astrocytes and *β-ACTIN* for neurons and microglia using 2-ΔCT calculation method; **P<0.01, ***P<0.001, ****P<0.0001. Data are mean ± SEM; *n* = 6 per group.

Hypoxic stress was associated with a progressive augmention of *AQP* diversity in neuronal and glial cell lines, as measured in a time series following restoration of normoxia (Figure 3 and Table 1). In SHSY5Y neurons, *AQP0, AQP3, AQP4, AQP5* and *AQP11* were elevated immediately after the termination of hypoxia (at 0 h normoxia). By 12 h after return to normoxia, *AQP5* and *AQP11* remained high, and transcript appeared for *AQP1*. By 24 h normoxia, the roster of expressed transcripts showed a cumulative expansion to include the initial classes *AQP0, AQP3, AQP4, AQP5* and *AQP11*, as well as *AQP4, AQP6, AQP7, AQP8* and *AQP9*, all at significantly increased levels as compared with controls (Figure 3B). In sum, the up-regulation of *AQP0* and *AQP11* emerged as a consistent theme for both neuronal and glial cell types across hypoxia and LPS stress conditions.

**Table 1:** Summary table of the classes of *AQP* genes that showed up-regulated transcript levels in response to LPS (10 and 100 ng/ml at 24 h) or hypoxia (5 min, followed by 12 and 24 h normoxia), measured in SHSY5Y neurons and 1321N1 astrocytes, and determined relative to levels in matched non-stressed control cells

Cell line	LPS	Hypoxia
SHSY5Y	*AQP 0, 1, 4, 5, 9, 11*	*AQP 0, 1, 3, 4, 5, 6, 7, 8, 9, 11*
1321N1	*AQP 0, 11*	*AQP 0, 1, 2, 3, 11*

### AQP0 and AQP11 protein expression in 1321N1 astrocytes and SHSY5Y neurons in response to LPS stress

**Figure 4:**
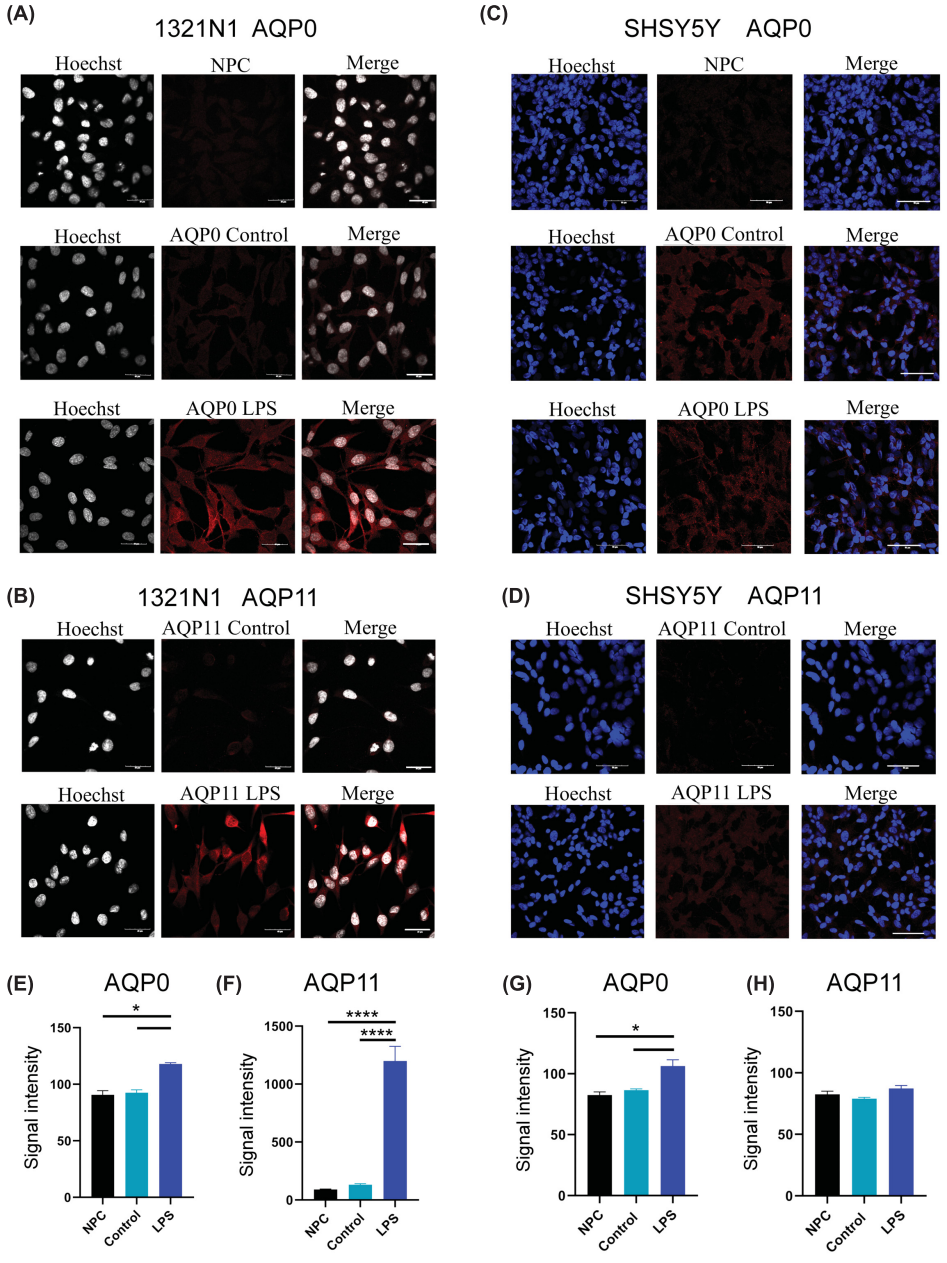
LPS-induced expression of AQP0 and AQP11 in 1321N1 astrocytes and SHSY5Y neurons Astrocytes (**A**) and neurons (**C**) immunolabeled for AQP0 (red), shown at 24 h without (control) or with 100 ng/ml LPS treatment; NPC, no primary antibody control. Immunolabeling of astrocytes (**B**) and neurons (**D**) for AQP11 (red), shown at 24 h without (control) or with 100 ng/ml LPS treatment. Hoechst nuclear staining is white for astrocytes and blue for neurons. Scale bars are 40 μM for astrocytes and 50 μM for neurons. Total fluorescence signal intensities for AQP0 in astrocytes (**E**), AQP11 in astrocytes (**F**), AQP0 in neurons (**G**) and AQP11 in neurons (**H**) were quantified from merged images in HALO using the Area-Quantification FL V.2.1.3 program (see Materials and methods for details), and plotted as histograms with mean ± SEM levels for two samples per treatment group; *P<0.05, ****P<0.0001.

The intriguing up-regulation of transcripts for *AQP0* and *AQP11* in response to neuropathological stressors prompted ICC characterization of protein expression patterns in 1321N1 and SHSY5Y cells (Figure 4). LPS was selected as the stressor, taking advantage of its focused effect in up-regulating AQP0 and AQP11 specifically. The glial line showed robust peroxiporin up-regulation after LPS stress, contrasting with subtle effects in the neuronal line. 1321N1 astrocytes treated with LPS (100 ng/ml, at 24 h) showed strong protein signals for both AQP0 and AQP11 (Figure 4A,B). Differentiated SHSY5Y neurons under the same LPS treatment showed low but detectable expression of AQP0 but minimal or no signal for AQP11 (Figure 4C,D).

ICC signal levels were quantified using the HALO system and data were compiled for statistical analyses (Figure 4E–H). LPS increased protein expression in the astrocyte cell line by 0.3 fold for AQP0 and 10-fold for AQP11(Figure 4E,F), as compared with non-stressed negative control cells. In differentiated neurons, LPS increased AQP0 only by 0.2-fold and did not appreciably change AQP11 levels (Figure 4G,H), which remained low despite the increased *AQP11* transcript (as described above in Figure 2). The patterns of AQP expression induced by stress showed cell-specific differences. The lack of an increase in AQP11 protein in the neuronal line, despite LPS-induced increases in AQP11 transcript, might suggest that protein synthesis of AQP11 requires longer than 24 h in this cell type. Slower protein translation also could be expected to delay the proposed protective effects of AQP11 in the neuronal cell type; this idea remains to be addressed in future work.

### AQP0 and AQP11 are both localized in the membrane of 1321N1 astrocytes

**Figure 5:**
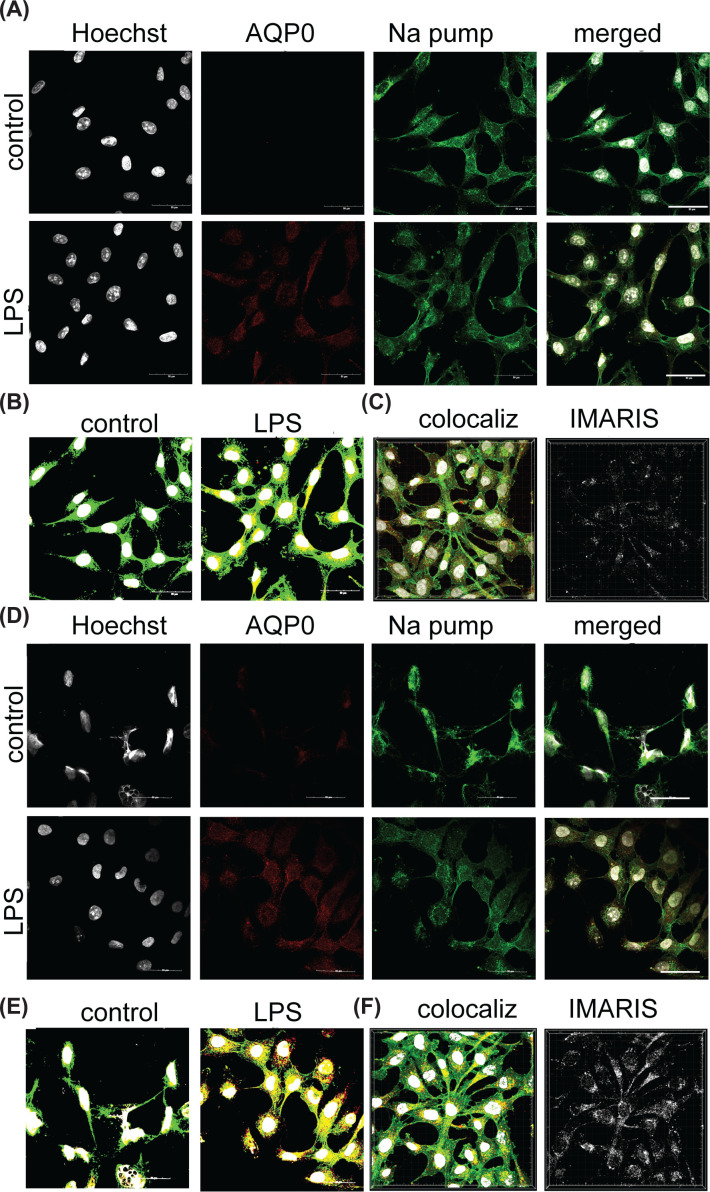
AQP0 and AQP11 channels up-regulated by LPS in 1321N1 astrocytes are located in plasma membrane Double-immunolabeling results show 1321N1 astrocytes with anti-Na^+^-K^+^-ATPase (‘Na pump’, green; plasma membrane marker), and anti-AQP (red) for AQP0 (**A–C**) or AQP11 (**D–F**). Top rows in (A) and (**D**) show non-LPS controls; bottom rows show treatments with 100 ng/ml LPS. Left columns in A and D show nuclear stain with Hoechst (white). Merged images analyzed by HALO (B,E) illustrate double labeling. Signal intensities for Na pump colocalization with AQP0 (C) or AQP11 (F) were measured with IMARIS in 100 ng/ml LPS-stimulated 1321N1 astrocytes using Z-stack images (‘colocaliz’; left images in C and F); colocalization scores are depicted in white (right images, C and F) as measured using IMARIS. Scale bars are 50 μm.

Double-immunostaining of 1321N1 astrocytes with antibodies to AQP0 or AQP11 and to the membrane pump Na^+^-K^+^-ATPase was used to assess plasma membrane localization (Figure 5). AQP0 was not evident in the baseline condition (Figure 5A, row 1), but was up-regulated by 100 ng/ml LPS (Figure 5A, row 2), with channels colocalizing with the plasma membrane marker as determined by HALO imaging (Figure 5B) and confocal Z-stack analysis using IMARIS (Figure 5C). Similarly, AQP11 was present in the plasma membrane of 1321N1 astrocytes after 100 ng/ml LPS stimulation (Figure 5D), confirmed with both HALO imaging (Figure 5E) and IMARIS analysis (Figure 5F). Plasma membrane levels of AQP0 and 11 both increased in signal intensity under 100 ng/ml LPS in the glial cells as compared with non-LPS controls.

### 1321N1 astrocytes express AQP0 and AQP11 in the endoplasmic reticulum

**Figure 6:**
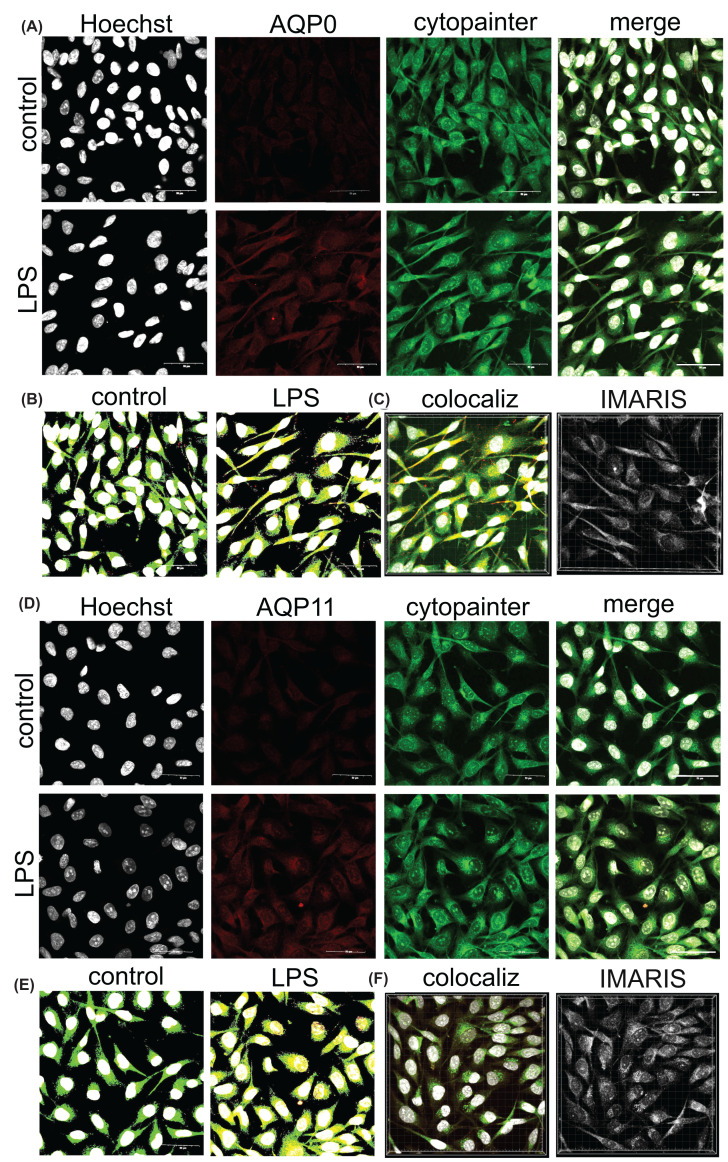
Increased levels of AQP0 and AQP11 channels in 1321N1 astrocytes at 24 h after LPS as compared with non-LPS controls Double labeling of 1321N1 astrocytes used the endoplasmic reticulum marker, cytopainter (green; ER marker) and anti-AQP antibodies visualized with anti-rabbit secondary (red) for AQP0 (**A–C**) or AQP11 (**D–F**). Top rows in (A) and (D) show non-LPS controls; bottom rows show treatments with 100 ng/ml LPS. Left columns in A and D show nuclear staining with Hoechst (white). Merged images analyzed by HALO (B,E) illustrate double labeling. Signal intensities for cytopainter colocalization with AQP0 (C) or AQP11 (F) were measured with IMARIS in 100 ng/ml LPS-stimulated 1321N1 astrocytes using Z-stack images (‘colocaliz’; left images in C and F); colocalization scores are depicted in white (‘IMARIS’; right images, C and F) as measured using IMARIS software. Scale bars are 50 μm.

Co-staining of the 1321N1 astrocytes with anti-AQP0 or anti-AQP11 antibodies and an endoplasmic reticulum marker, Cytopainter, (Figure 6) showed ER localization of AQP0 (Figure 6A) confirmed by HALO (Figure 6B) and IMARIS Z-stack analysis (Figure 6C). AQP11 was also expressed in ER (Figure 6D), showing increases in both fluorescence intensity and level of colocalization in ER after LPS stress (Figure 6E). These data demonstrated AQP0 and AQP11 proteins are expressed and localized in both plasma membrane (Figure 5) and ER (Figure 6) in 1321N1 astrocyte cells, and that channel levels in glial cells, particularly for AQP11, are exacerbated by inflammatory stress.

### Differentiated SHSY5Y neurons express AQP0 in endoplasmic reticulum in response to LPS

**Figure 7:**
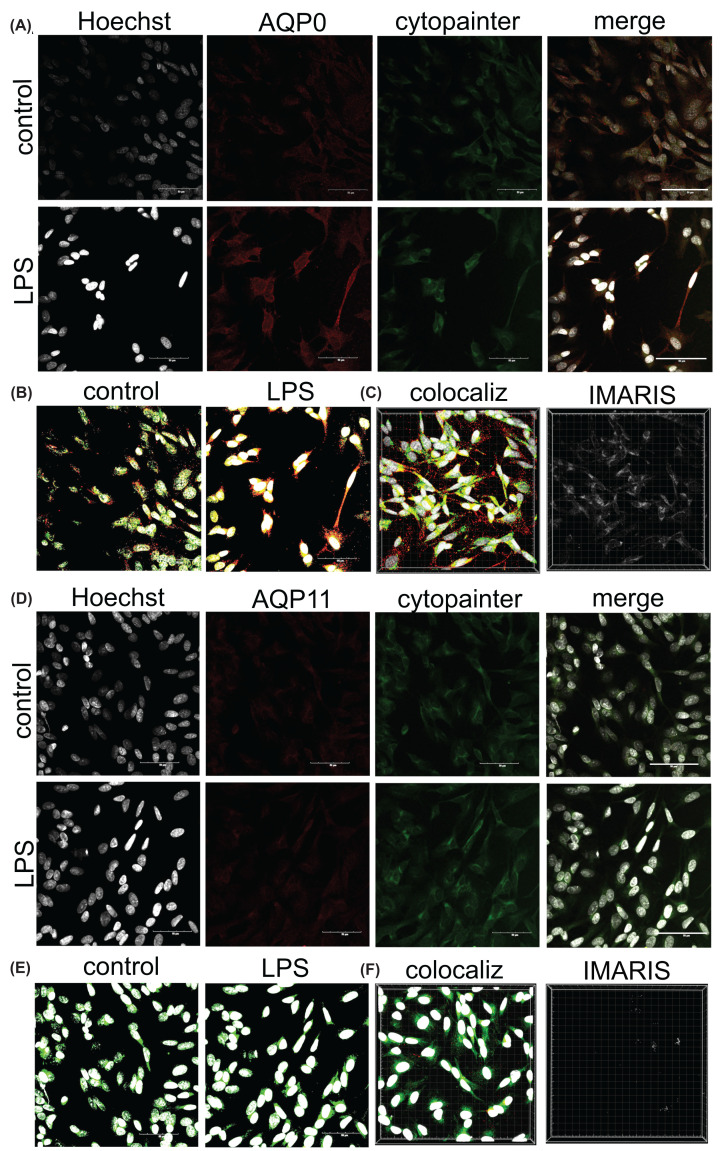
Increased levels of AQP0 but not AQP11 channels in SHSY5Y neurons at 24 h after LPS as compared with non-LPS controls Images show double labeling of SHSY5Y neurons with cytopainter (green; ER marker), and anti-AQP (red) for AQP0 (**A–C**) or AQP11 (**D–F**). Top rows in (A) and (D) are non-LPS controls; bottom rows are treatments with 100 ng/ml LPS. Left columns in A and D show nuclear stain with Hoechst (white). Merged images analyzed by HALO (B,E) illustrate double labeling. Signal intensities for cytopainter colocalization with AQP0 (C) or AQP11 (F) were measured with IMARIS in LPS-stimulated SHSY5Y neurons using Z-stack images (‘colocaliz’; left images in C and F); colocalization scores depicted in white (right images, C and F) were measured using IMARIS. Scale bars are 50 μm.

In the ER, AQP0 showed substantial colocalization with the marker Cytopainter (Figure 7) after LPS-induced stress (Figure 7A) in differentiated SHSY5Y neurons, as shown by analyses with HALO (Figure 7B) and IMARIS (Figure 7C). Consistent with data above (see Figure 4), AQP11 protein was not evident in neurons under control or LPS-stress conditions at 24 h (Figure 7D), thus no colocalization with either the ER marker (Figure 7E,F) or the plasma membrane markers was evident. Unlike the 1321N1 astrocytes, which showed rapid up-regulation of both AQP0 and AQP11 transcription and translation, the differentiated SHSY5Y neurons only showed a rapid response for AQP0. This induced AQP0 was predominantly intracellular in SHSY5Y neurons. The lack of an IMARIS signal suggested minimal if any colocalization with Na^+^-K^+^-ATPase, indicating AQP0 wasnot found at appreciable levels in plasma membrane at the time of experimental evaluation (Supplementary Figure S3).

### AQP0 and AQP11 are linked to peroxiporin functions in 1321N1 astrocytes

**Figure 8:**
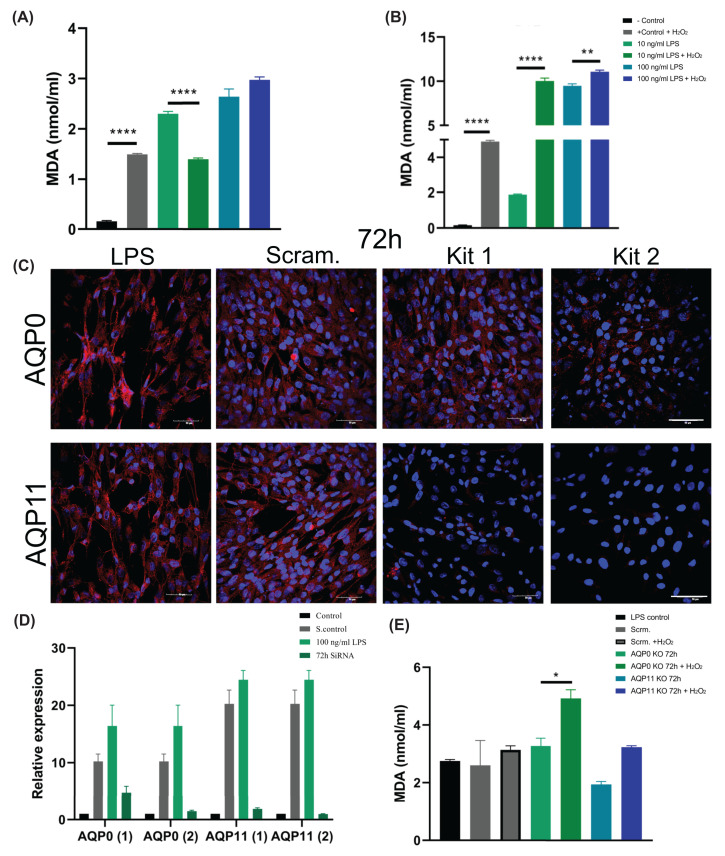
Novel peroxiporin function of AQP0 in 1321N1 astrocyte-like cells MDA levels (nmol/ml) in (**A**) 1321N1 astrocytes and (**B**) BV2 microglia in control and LPS stimulation with and without 5 μM H2O2; *n*=8. (**C**) Immunocytochemistry of 1321N1 astrocytes incubated with 2 siRNA kits for AQP0 (top row) and AQP11 (bottom row) for 72 h; scrambled siRNA kits served as an experimental control; LPS stimulation served as a positive control. (**D**) Expression of *AQP0* and *AQP11* relative to *GAPDH* in astrocytes after siRNA knockdown, using two kits each with scrambled siRNA kit as a control. RNA extraction was done at 72 h of siRNA treatment, after a time course that included replacement of transfection media at 48 h and 100 ng/ml LPS stimulation for 24 h. Approximately 100 ng/ml LPS-stimulated cells served as a positive control; no treatment was a negative control; *n*=6. (**E**) MDA levels in astrocytes following scrambled, AQP0 and 11 siRNA treatments (using kits 2) at 72 h, after 10 ng/ml LPS stimulation with and without H_2_O_2_ as indicated; *P<0.05, **P<0.01, ***P<0.001, ****P<0.0001; Mean ± SEM; *n*=8.

AQP0 in the eye and AQP11 in adipose tissue serve essential peroxiporin functions [63,64]. To evaluate a potential role in peroxide transport for these AQP classes in 1321N1 astrocytes, levels of lipid peroxidation after brief exposures to H_2_O_2_ were quantified using the MDA assay (Figure 8). Non-LPS control 1321N1 glial cells challenged with a brief pulse of H_2_O_2_ (5 min at 3 µM) displayed high MDA levels, as expected for stress-induced peroxidation. In contrast, the 1321N1 cells that had been exposed to LPS 24 h earlier showed greatly attenuated levels of lipid peroxidation in response to the brief pulse of H_2_O_2_, and no effect of H_2_O_2_ after pretreatment with 100 ng/ml LPS (Figure 8A), suggesting protection had been established. In contrast, microglial cells showed significant elevations in MDA levels after single pulses of H_2_O_2_ with or without pretreatment with 10 or 100 ng/ml LPS stimulation (Figure 8B). The protection from lipid peroxidation in glia coincided with the up-regulation of perioxiporin expression levels in the glial cells, whereas microglia lacking the AQP up-regulation response were substantially more impacted by oxidative stress.

To assess the direct roles of the peroxiporins in the apparent protective response which developed after LPS pretreatment, cultures of 1321N1 astrocytes were prepared by siRNA knockdown of AQP0 or AQP11, compared with scrambled siRNA and untreated controls (Figure 8C,D). Two siRNA kits each for AQP0 and AQP11 were assessed by qPCR and immunocytochemistry to establish the efficacy and optimal durations of siRNA treatments. Parallel treatments with the scrambled siRNA kit and untreated cells served as controls. The maximal knockdown for both AQPs was seen at 72 h post-transfection. For AQP0, kit 2 was more effective than kit 1; for AQP11 both kits were effective, and kit 2 was selected for further assays (Figure 8C,D). Strikingly, AQP knockdown removed the protective effect of LPS pretreatment, rendering the 1321N1 astrocytes vulnerable to lipid peroxidation caused by a brief H_2_O_2_ exposure. No effects of the scrambled control on MDA levels were seen with or without H_2_O_2_. The loss of protection in glia after AQP0 knockdown yielded a clear increase in vulnerability, and a similar but not statistically significant trend was observed after AQP11 knockdown (Figure 8D). These results are consistent with protective roles for these peroxiporin channels, enabled by dynamic up-regulation of selected AQP expression in response to prior stressor challenges.

## Discussion

Published transcriptomic analyses of classes of aquaporins in the human brain uncovered unexpected subtypes of AQPs that were shown to function in other tissues as peroxiporins [14], including the lens aquaporin AQP0 in the eye [65] and endoplasmic reticulum channel AQP11 in the kidney [66]. Dynamic regulation of mammalian brain AQPs in response to stressors was proposed based on RNAseq analyses that confirmed differential changes in human *AQP* expression patterns with age and neuropathology [14], but the AQP localization and sensitivity to environmental stimuli remained unknown. Work here used differentiated SHSY5Y human neuronal and 1321N1 glial cell lines as model systems for investigating AQP0 and AQP11 involvement in brain cell stress responses.

The main outcome of this work was the demonstration that transcript and protein levels of AQP0 and AQP11 were enhanced after hypoxic or LPS stress in both glia and neuronal cell lines. The themes of up-regulating *AQP0* and *AQP11* in response to cellular stress were consistent with proposed compensatory or protective roles for these channels, potentially relevant in conditions such as Alzheimer’s disease, where oxidative stress is prominent [67]. Aquaporin gene expression responses depended on the stressor conditions. The patterns of *AQP* up-regulation were more complex after hypoxic stress than after LPS. Transcript levels of *AQP1* increased after hypoxia but not LPS in both glial and neuronal cell lines. AQP0 is a peroxiporin channel expressed in lens cells of the eye [63]. AQP1 is a peroxiporin, as well as a dual water and ion channel [19,28,68–70] that influences neural crest cell migration in early central nervous system development and dorsal root sensory signal transduction [71,72]. Transcript levels for *AQP3* were shown here to increase after hypoxia but not LPS in both cell lines. AQP3 is an aquaglyceroporin [23] identified previously in post-mortem human hippocampus [14], increased in rat astrocytes and neurons under ischemic stress [29], which could enhance metabolic activity by glycerol uptake in response to hypoxia [73]. The transcript levels for *AQP4* in response to hypoxia or LPS stress increased in SHSY5Y neurons but not 1321N1 astrocytes. The lack of an astrocyte response for *AQP4* in the 1321N1 model contradicts results of studies in brain tissue [74,75] and could reflect the absence of environmental factors such as endfoot contact with endothelial cells, essential for fluid homeostasis and glymphatic function [2,3,5,13]. Transcripts for *AQP5* and *AQP9* were increased in neuronal but not astroglial model cell lines after hypoxia or LPS stress. AQP5 is a peroxiporin [63], shown in prior work from our group to increase at the transcript level in human hippocampus and parietal cortex portmortem samples [14]. Prior work using an RNAseq database based on human brain samples found *AQP5* transcript levels were elevated in the human hippocampus with age [14]. Work here determined *AQP5* in addition to other peroxiporins *AQP6* and *AQP8* [25,26] were up-regulated in SHSY5Y neurons but not 1321N1 astrocytes under stress, suggesting the transcriptomic data might have reflected responses predominantly in neuronal rather than astroglial populations in the affected brain regions, a prediction that remains to be tested. In SHSY5Y neurons, we found *AQP7* increased under hypoxic stress. AQP7 and AQP9 are aquaglyceroporins that provide metabolic support [76,77]. Based on its subcellular localization in brain mitochondrial inner membranes, AQP9 in mammalian CNS astrocytes and midbrain dopaminergic neurons has been proposed to provide fluxes of lactate and other metabolites to adapt to changes in metabolic status [78]; AQP7 might serve similar roles. AQP9 is permeable to a broad spectrum of substrates including purines, pyrimidines, and lactate, in addition to water and glycerol. An age-associated decrease in both transcript and protein levels of AQP9 was described in hippocampus and cerebral cortex in a transgenic AD mice model; in that study siRNA knockdown increased the neurotoxic consequences of amyloid β(1-40) exposure [79], consistent with a protective role for AQP9 in neurons.

Results here showed that AQP0 proteins are present in the plasma membrane and endoplasmic reticulum in human 1321N1 and differentiated SHSY5Y cells, and that AQP11 is present in both the endoplasmic reticulum and plasma membrane of 1321N1 astrocytes, increased after LPS stimulation, but not detectable in SHSY5Y neurons. The peroxiporin functions of AQP0 and AQP11 were corroborated by results here showing protection from lipid peroxidation in 1321N1 astrocytes, an effect that was compromised by siRNA knockdown of AQP0 and showed an interesting comparable trend for AQP11 that merits exploration in future studies.

**Figure 9:**
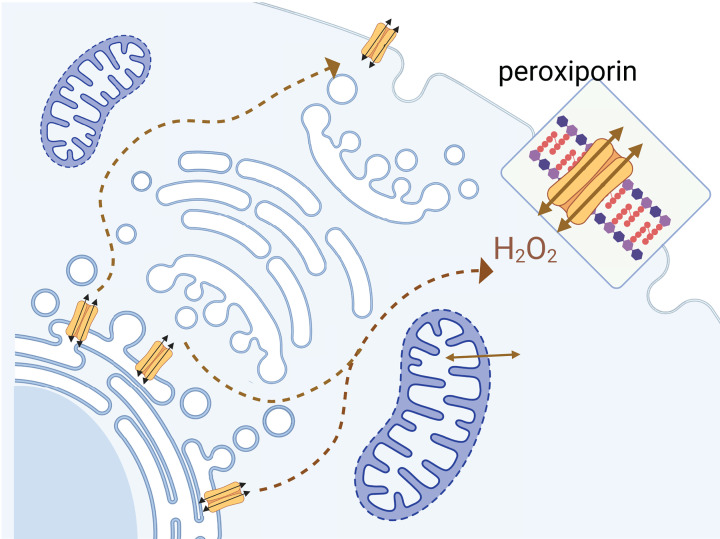
Peroxiporin pathway enabling the efflux of the oxidative stressor hydrogen peroxide from cells Schematic illustration of the role of the peroxiporin AQP11 in conferring protection from elevated hydrogen peroxide (H_2_O_2_) levels, enabled by high levels of expression of AQP11 channels in endoplasmic reticulum, with additional expression in plasma membrane. Export of H_2_O_2_ from intracellular organelles into the cytoplasm followed by removal from the cytoplasm to extracellular fluid compartments is proposed to offset consequences of oxidative stressor accumulation associated with metabolic activity. Graphic generated using Biorender, Publication Agreement # AU26IJ5C1E.

AQPs are increasingly being recognized for mediating functions beyond the passive flow of water across biological membranes [80]. Development of small-molecule AQP inhibitors is anticipated to provide insights into basic biology and new treatments for a wide range of AQP-associated disorders [17,18]. Classes of AQPs conduct a wide range of solutes, including urea, glycerol, gases such as carbon dioxide and nitric oxide, ammonia, and hydrogen peroxide [81]. Aquaporin-facilitated H_2_O_2_ diffusion across biological membranes controls aspects of membrane signaling in diverse organisms [82]. H_2_O_2_ permeability has been suggested to be a broad feature of many classes of water-permeable AQPs, adding functional capacities not predicted by the intrasubunit pore diameter alone [83]. The initial concept that H_2_O_2_ permeability across endoplasmic reticulum relied just on simple diffusion was challenged by work showing AQP-mediated H_2_O_2_ transport across endoplasmic reticulum membrane was necessary for signal transduction [84]. Evidence for permeation of H_2_O_2_ through plant Arabidopsis TIP1;1 and TIP1;2 and human AQP8 channels was confirmed by screening yeast strains sensitive to oxidative stress [85]. AQP0 channels in the lens of the eye [86] have complex roles in biomechanical maintenance of lens fiber structural integrity as well as peroxiporin transport [63,86]. AQP11 present in endoplasmic reticulum in kidney and visceral adipocytes [27] is linked to redox homeostasis and signaling [64]. Peroxiporin pathways enabling the efflux of oxidative stressors such as hydrogen peroxide are consistent with high levels of expression of AQP11 in endoplasmic reticulum, and additional channel expression in plasma membrane, as illustrated in Figure 9, in order to allow export from intracellular organelles into cytoplasm followed by removal from the cytoplasm to extracellular fluid compartments to offset oxidative stress. Review articles have noted the impressive range of functional roles for AQP channels being discovered in mammalian health and disease [17–19,87–90].

Emerging evidence points to an intriguing role for diverse classes of peroxiporins as protective mechanisms in tissues throughout the body. Mouse AQP0, AQP1 and AQP5 peroxiporins facilitated transmembrane H_2_O_2_ transport in the lens of the eye, protecting against cataract formation [63]. AQP1 in human visceral adipocytes was suggested to serve as a compensatory mechanism to alleviate endoplasmic reticulum stress in obesity [91]. AQP1 expression levels in human and mouse cardiac myocytes correlated with disease severity in hypertrophic hearts; located in cardiac plasma membrane, AQP1-mediated influx of extracellular H_2_O_2_ through the intrasubunit water pore resulted in activation of oxidant-sensitive kinases, driving hypertrophic responses that were counteracted by the AQP1 water pore blocker bacopaside II [28]. Higher levels of expression of peroxiporins AQP1, AQP3 and AQP5 in colon cancer cell lines correlated with better protection from oxidative insults, even when the background activity of antioxidant defense system components remained unchanged [92]. Human AQP5 was shown to be a highly efficient peroxiporin, characterized in transformed yeast cells exposed to oxidative stress, and in human pancreatic cancer cells [93]. AQP3, AQP5 and AQP8 contribute to ferroptosis by regulating the permeability of extracellular H_2_O_2_ [94]. Genetic knockout of AQP5 promoted retention of H_2_O_2_ in eye lens and worsened the loss of transparency as compared with wild-type under hyperglycemic conditions, suggesting a protective role for AQP5 in oxidative stress [63]. Overexpression of AQP8 in mitochondria and plasma membrane increased proliferation and insulin content in the pancreatic cell line RINm5F, with transport of H_2_O_2_ confirmed with the sensor HyPer7.2; at low levels, H_2_O_2_ promoted pancreatic β-cell growth, but at high levels impaired insulin secretion, suggesting possible pathological mechanism relevant to diabetes mellitus [95]. In motile sperm, AQP8 has a primary role in removal of hydrogen peroxide [96]. AQP9 is permeable to H_2_O_2_, as well as glycerol and water, beneficial in liver regeneration. Impaired H_2_O_2_ transport across plasma membranes of cultured AQP9^−/−^ hepatocytes resulted in H_2_O_2_ accumulation and oxidative injury, whereas overexpression of AQP9 or AQP3 in AQP9^−/−^ mice fully rescued liver regeneration, oxidative protection and glucose metabolism [97]. Up-regulation of various AQP classes in inflammatory conditions is essential for immune cell migration and viability [98]. Optical probes targeted to organelles showed AQP11 localized in the endoplasmic reticulum efficiently enabled fluxes of H_2_O_2_ [99], needed for transferring H_2_O_2_ from mitochondria to the ER [100], a possible explanation for localization observed in the ER of 1321N1 astrocytes in the present study. Insights into the diverse roles of peroxiporins as protective mechanisms are emerging in multiple research areas [82,101–103].

Work here provides the first lines of experimental evidence for the expression of AQP0 and AQP11 in 1321N1 astrocytes and a protective function for AQP0 in reducing lipid peroxidation. Coupled with previous work from our group that identified transcript levels of peroxiporins in the aging and Alzheimer’s brain [14], stress-induced expression of AQP0 and AQP11 appears to be emerging as an important general response mechanism. Similar though not identical patterns of changes in mRNA and protein levels of AQPs as described here have been reported in tissue samples from human patients with temporal lobe epilepsy based on microarray and ELISA analyses [104]. Protective roles for peroxiporins in reducing oxidative stress-induced lipid peroxidation could be highly relevant across multiple neuropathologies. Peroxiporin facilitated clearance of H_2_O_2_ would be predicted to reduce OS and consequently reduce markers of stress such as lipid peroxidation. A testable concept for future work will be to investigate the role of H_2_O_2_ as a stimulus regulating levels of AQP expression. Peroxiporins might serve as stress detectors as well as responders. Other classes of AQP channels that were up-regulated in the neuronal cell line but not evaluated here also merit further investigation for possible roles in cellular homeostasis.

In conclusion, work here proposes that up-regulation of AQP0 and AQP11 expression could be an adaptive mechanism to reduce the toxic effects H_2_O_2_ accumulation, alleviating damage associated with neurodegenerative diseases such as Alzheimer’s disease, in which oxidative stress is prominent [67]. Our results highlight an expanding role for dynamic regulation of multiple classes of AQP channels in homeostasis and health, and open new paths for advancing understanding of aging and disease mechanisms in the human brain. Up-regulation and localization of peroxiporin channels is an adaptive mechanism which facilitates the movement of H_2_O_2_ between organelles, cytosol and extracellular fluid in brain glia and neurons. Patterns of AQP expression might correlate with neuronal vulnerability to oxidative stress and metabolic challenges [105,106]. Peroxiporins serving neuroprotective roles would be intriguing targets for the development of therapeutic interventions aimed at slowing progression of neuropathological disease conditions.

## Supplementary Material

Supplementary Figures S1-S3 and Table S1

## Data Availability

All supporting data and results are presented in the paper. Additional details are available on request from the corresponding author.

## Open Access

Open access for this article was enabled by the participation of University Of Adelaide in an all-inclusive *Read & Publish* agreement with Portland Press and the Biochemical Society under a transformative agreement with CAUL.

## Abbreviations

AD: Alzheimer’s disease
AQP: aquaporin
ICC: immunocytochemistry
LPS: lipopolysaccharide
MDA: malondialdehyde
qPCR: quantitative PCR
RA: retinoic acid
