# Supplementary material for: Protective roles of peroxiporins AQP0 and AQP11 in human astrocyte and neuronal cell lines in response to oxidative and inflammatory stressors
Source: Biosci Rep. 2024 Mar 22;44(3):BSR20231725. doi: 10.1042/BSR20231725 (PMC10965398; doi:10.1042/BSR20231725)
Supplement: Supplementary Figures S1-S3 and Table S1 [file BSR-2023-1725_supp.pdf]

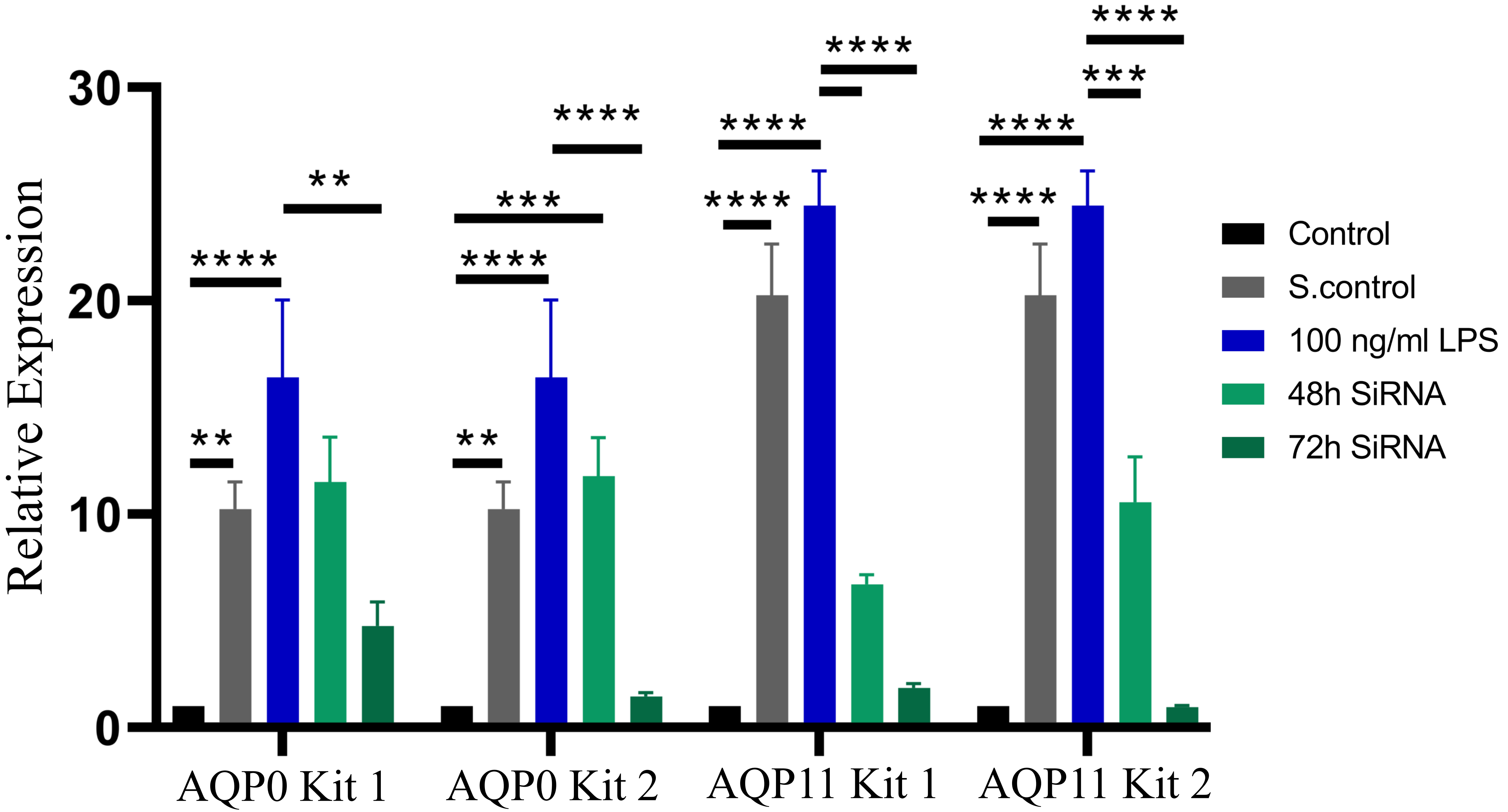

Relative Expression
30
20
10
0
AQP0 Kit 1
AQP0 Kit 2
AQP11 Kit 1
AQP11 Kit 2
Control
S.control
100 ng/ml LPS
48h SiRNA
72h SiRNA
**
****
**
***
****
**
****
****
****
****
****
****
****
***

Supplementary Figure S1: Relative transcript levels of *AQP0* and *AQP11* standardized to the reference gene *GAPDH* in astrocytes after siRNA knockdown treatments. Two siRNAs per construct and scrambled siRNA controls were tested at 48h and 72h. RNA from 1321N1 astrocytes was extracted at either 48h or 72h following 100 ng/ml LPS stimulation for 24h. 100 ng/ml stimulated cells served as a positive control, with no treatment as a negative control. **P<0.005, ***P<0.001, ****P<0.0001.

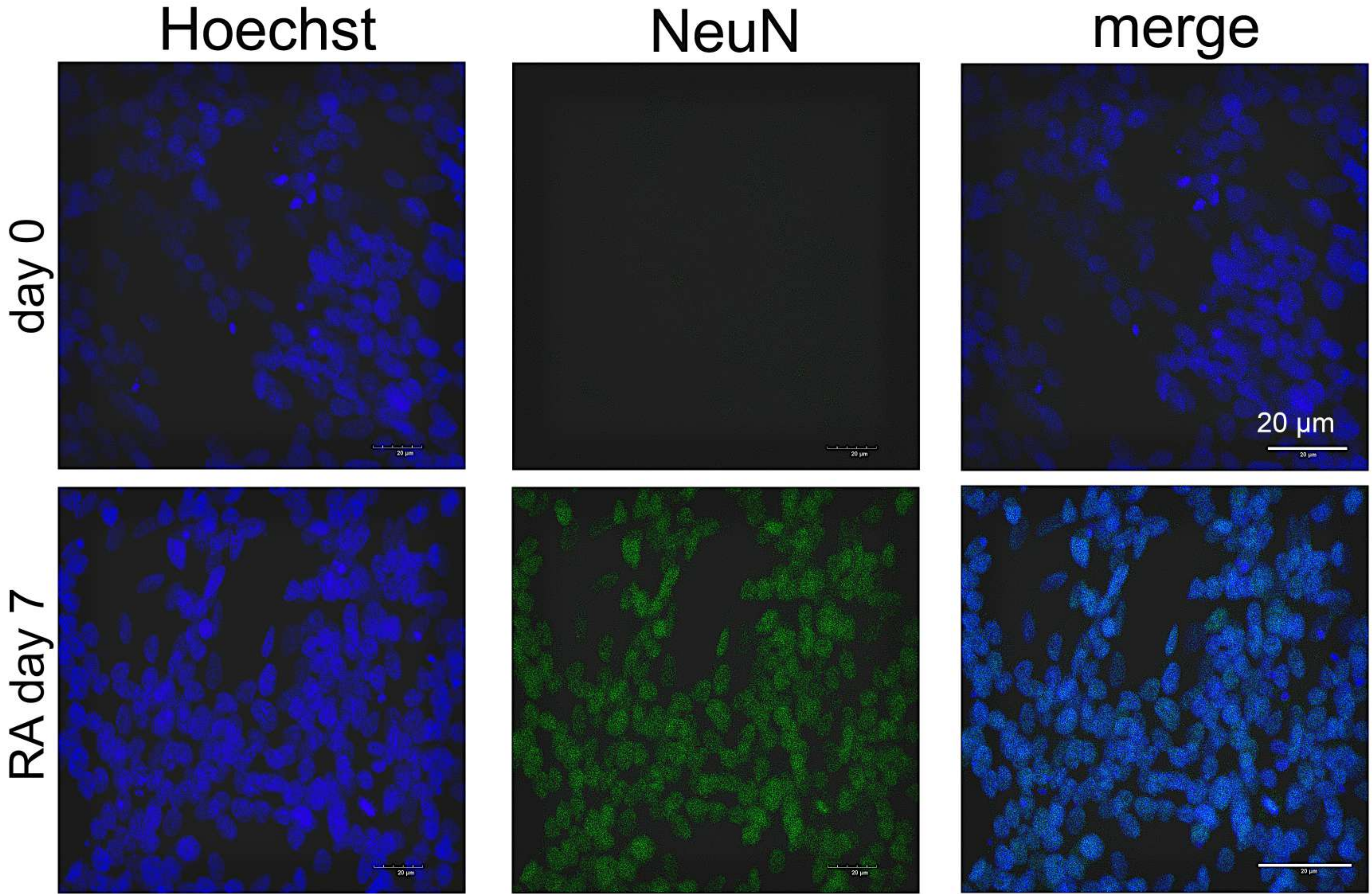
Hoechst
NeuN
merge
day 0
RA day 7
20 μm

Supplementary Figure S2: Neuronal marker confirmation of SHSY5Y differentiation. Differentiated neurons at day 7 in retinoic acid (RA) express the mature neuronal marker NeuN not seen in the undifferentiated neuroblastoma cells (day 0). NeuN (1:300; green) and Hoechst nuclear staining (1:1000; Blue). Secondary antibodies used were Alexa488 (1:1000; Green) and Alexa568 (1:1000; Red). Scale bar 20 µm.

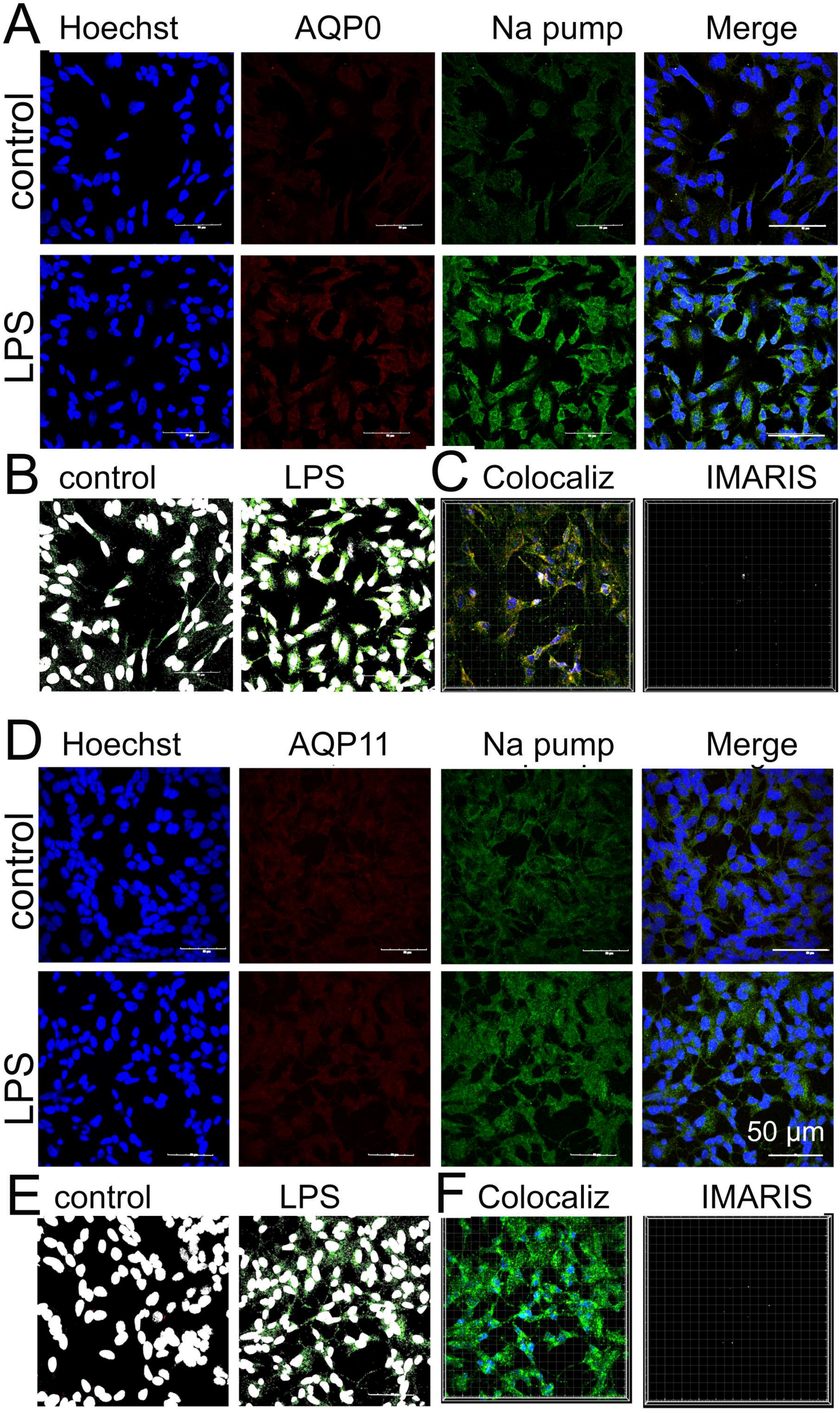
A
Hoechst
AQP0
Na pump
Merge
control
LPS
B
control
LPS
C
Colocaliz
IMARIS
D
Hoechst
AQP11
Na pump
Merge
control
LPS
50 μm
E
control
LPS
F
Colocaliz
IMARIS

Supplementary Figure S3: AQP0 and AQP11 channels are not located in the plasma membrane of SHSY5Y neurons under LPS stress. Double-immunolabeling results show SHSY5Y neurons with anti-Na^{+}-K^{+}-ATPase ("Na pump", green; plasma membrane marker), and anti-AQP (red) for AQP0 **(A-C)** or AQP11 **(D-F).** Upper rows in **(A)** and **(D)** show non-LPS controls; lower rows show treatments with 100 ng/ml LPS. Left columns in A and D show nuclear staining with Hoechst (blue). Merged images analyzed by HALO **(B, E)** illustrate double labeling. Signal intensities for Na pump colocalization with AQP0 **(C)** or AQP11 **(F)** were measured with IMARIS for 100 ng/ml LPS-stimulated SHSY5Y neurons using Z-stack images ("colocaliz"; left images in C and F); colocalization scores are depicted in white (right images, C and F) as measured using IMARIS. Scale bars are 50 µm.

Supplementary Table 1. Sequences of primers used for quantitative PCR amplification of signals for all classes of human AQPs 0 to 12, and reference genes GADPH and ß-actin.

gene	direction	sequence	product size (bp)
AQP0	Forward<br>Reverse	5’CTAGCACTCAACACGTTGCAC<br>3’AGGATTCATCGCCTGCACCAG	210
AQP1	Forward<br>Reverse	5’CCTTGGACACCTCCTGGCTATTG<br>3’CTTCACGCGGTCTGTGAGGT	199
AQP2	Forward<br>Reverse	5’ATGGCGTTTGGCTTGGGTAT<br>3’GATGTCTGCTGGCGTGATCT	200
AQP3	Forward<br>Reverse	5’ACCAGCTTTTTGTTTCGGGC<br>3’AGGCTGTGCCTATGAACTGGT	111
AQP4	Forward<br>Reverse	5’CCTCGCTGGTGGCCTTTATGA<br>3’GTCTTTCCCCTTCTTCTCCTCTCC	207
AQP5	Forward<br>Reverse	5’CCACCTTGTCGGAATCTACTT<br>3’TTTGATGATGGCCACACGC	205
AQP6	Forward<br>Reverse	5’CCATCATCATTGGGAAGTTCACAG<br>3’GCGTAGGCTGTTTCACACACTCTC	251
AQP7	Forward<br>Reverse	5’CACAGGCGGTCCACCC<br>3’TCATGAACTCGGCCAGGAAC	109
AQP8	Forward<br>Reverse	5’ATGTCTGGTCGAACTGCTGG<br>3’CAGTACGGGAGGAGCATCAC	231
AQP9	Forward<br>Reverse	5’ATCGTGGGAGAAAATGCAAC<br>3’CAATAATCAGGAGGCCGATG	196
AQP10	Forward<br>Reverse	5’TGCAGTGACAGTGTGCCTAT<br>3’TGGGTGAGGAGCATGAGTACA	178
AQP11	Forward<br>Reverse	5’GCTTTGGCACTTTCGCTACA<br>3’TGCAGCCGGTGTTTTCCATA	253
AQP12A	Forward<br>Reverse	5’CCTGCTCTCCTGCTCTTCC<br>3’AGAGACTGCTCGGCCATGA	102
AQP12B	Forward<br>Reverse	5’TCTTTGCCACCTTCACCCTC<br>3’GTCCTCATCTCCAGGAAGCA	136
GADPH	Forward<br>Reverse	5’TCACCAGGGCTGCTTTTAAC<br>3’TGACGGTGCCATGGAATTTG	129
ß-actin	Forward<br>Reverse	5’CTTCGCGGGCGACGAT<br>3’CCACATAGGAATCCTTCTGACC	104
